# Excerpts from the 1st international NTNU symposium on current and future clinical biomarkers of cancer: innovation and implementation, June 16th and 17th 2016, Trondheim, Norway

**DOI:** 10.1186/s12967-016-1059-6

**Published:** 2016-10-19

**Authors:** Ana I. Robles, Karina Standahl Olsen, Dana W.T. Tsui, Vassilis Georgoulias, Jenette Creaney, Katalin Dobra, Mogens Vyberg, Nagahiro Minato, Robert A. Anders, Anne-Lise Børresen-Dale, Jianwei Zhou, Pål Sætrom, Boye Schnack Nielsen, Michaela B. Kirschner, Hans E. Krokan, Vassiliki Papadimitrakopoulou, Ioannis Tsamardinos, Oluf D. Røe

**Affiliations:** 1 Laboratory of Human Carcinogenesis, National Cancer Institute, NIH, Bethesda, USA; 2 Department of Community Medicine, UiT The Artic University of Norway, Tromsø, Norway; 3 Department of Pathology and Center for Molecular Oncology, Memorial Sloan Kettering Cancer Center, New York, USA; 4 Department of Medical Oncology, School of MedicineUniversity of Crete, Heraklion, Greece; 5 National Centre for Asbestos Related Disease, University of Western Australia, Perth, Australia; 6 Division of Pathology, Department of Laboratory Medicine, Karolinska Institutet, Karolinska University Hospital, Stockholm, Sweden; 7 Department of Clinical Medicine, Institute of Pathology, Aalborg University Hospital, Aalborg University, Aalborg, Denmark; 8 Department of Immunology and Cell Biology, Graduate School of Medicine, Kyoto University, Kyoto, Japan; 9 Department of Pathology, Johns Hopkins University, Baltimore, USA; 10 Department of Cancer Genetics, Institute for Cancer Research, Oslo University Hospital, The Norwegian Radium Hospital, Oslo, Norway; 11 Department of Molecular Cell Biology & Toxicology, Cancer Center School of Public Health, Nanjing Medical University, Nanjing, People’s Republic of China; 12 Department of Computer and Information Science, NTNU, Trondheim, Norway; 13 Molecular Histology Bioneer A/S, Hørsholm, Denmark; 14 Division of Thoracic Surgery, University Hospital Zurich, Zurich, Switzerland; 15 Department of Cancer Research and Molecular Medicine, Norwegian University of Science and Technology (NTNU), Trondheim, Norway; 16 Department of Thoracic/Head and Neck Medical Oncology, MD Anderson, Houston, USA; 17 Department of Computer Science, University of Crete, Heraklion, Greece; 18 Cancer Clinic, Department of SurgeryLevanger Hospital, Nord-Trøndelag Hospital Trust, Levanger, Norway; 19 Department of Clinical Medicine, Clinical Cancer Research Center, Aalborg University Hospital, Aalborg, Denmark

**Keywords:** Bioinformatics, Circulating biomarkers, Circulating tumor cells, DNA repair, Early detection, Early diagnosis

## Abstract

The goal of biomarker research is to identify clinically valid markers. Despite decades of research there has been disappointingly few molecules or techniques that are in use today. The “1st International NTNU Symposium on Current and Future Clinical Biomarkers of Cancer: Innovation and Implementation”, was held June 16th and 17th 2016, at the Knowledge Center of the St. Olavs Hospital in Trondheim, Norway, under the auspices of the Norwegian University of Science and Technology (NTNU) and the HUNT biobank and research center. The Symposium attracted approximately 100 attendees and invited speakers from 12 countries and 4 continents. In this Symposium original research and overviews on diagnostic, predictive and prognostic cancer biomarkers in serum, plasma, urine, pleural fluid and tumor, circulating tumor cells and bioinformatics as well as how to implement biomarkers in clinical trials were presented. Senior researchers and young investigators presented, reviewed and vividly discussed important new developments in the field of clinical biomarkers of cancer, with the goal of accelerating biomarker research and implementation. The excerpts of this symposium aim to give a cutting-edge overview and insight on some highly important aspects of clinical cancer biomarkers to-date to connect molecular innovation with clinical implementation to eventually improve patient care.

## Background

The “1st international NTNU symposium on current and future clinical biomarkers of cancer: innovation and implementation”, was held June 16th and 17th 2016, at the knowledge center of the St. Olavs Hospital in Trondheim, Norway, under the auspices of the Norwegian University of Science and Technology (NTNU) and the HUNT biobank and research center. The Symposium attracted approximately 100 attendees and invited speakers from 12 countries and 4 continents. Senior researchers and young investigators presented, reviewed and vividly discussed important new developments in the field of clinical biomarkers of cancer, with the goal of accelerating biomarker research and implementation.

Inspiration for arranging this Symposium in Trondheim came from worldwide rapid developments in the biomarker field and current research based on the Norwegian Nord-Trøndelag Health Study (HUNT), a population study founded in 1986 that has evolved to become a Biobank and Research Center under the NTNU (http://www.mensxmachina.org/cancer_biomarker_hunt/index.html). The center is situated in Levanger and houses hundreds of clinical data variables, serum and DNA from about 120,000 people that are accessible for researchers. The meeting highlighted the unique potential and growing network around this resource, as well as the necessity of discussing clinical cancer biomarkers and their implementation in a broad forum.

The Symposium served to remind us that cancer is a collection of very heterogeneous diseases, making molecular sub grouping increasingly relevant for diagnosis and treatment. Since the discovery of the estrogen receptor in breast cancer, science has unraveled hundreds of clinically relevant diagnostic, prognostic, predictive and therapeutic molecular markers of cancer, including HER2, KRAS, EGFR, ALK, BRAF, CTL4, PD1 and PD-L1, circulating tumor cells, protein and gene signatures, and microRNAs. New epigenetic and metabolic markers are also entering the stage, increasing the potential as well as the complexity of targeted treatments for defined groups of cancer patients. However, few markers have passed all clinical development and validation phases and are actually in clinical use today. Following the initial discovery, the road taking a biomarker to clinical use is usually long and complex. Moreover, clinicians should always be aware of caveats that affect a biomarker’s broad applicability.

An iconic example is HER2/ERBB2 in breast cancer, initially discovered as a negative prognostic marker, it has now become a positive predictive marker due to the receptor’s “targetability” with trastuzumab, pertuzumab and other agents. This observation urges for the need for a set of accurate molecular diagnostic tests for each treatment strategy. For example, the potential of the immune system to attack cancer cells using the PD1/PD-L1 interaction, one of the most important breakthroughs that change the cancer therapeutic algorithm in recent years. However, about one-third of the cases respond to anti-PD1 monotherapy [1], and with the current cost of the treatment, picking the right patients will be of enormous value, not only for the patients but also the society. But are biomarkers for immune checkpoint therapy ready for use? Lastly, it is becoming apparent that detection of microRNAs or other molecules in circulation could be the first sign of early stage cancer, and could eventually become a blood test that saves thousands of lives. How far in the process are those tests? To reach the goals of true precision medicine and fulfill the Cancer Moonshot initiative [2] we need to valid these biomarkers and their accompanying diagnostic tests in the appropriate environment.

The excerpts of this symposium aim to give a cutting-edge overview and insight on some highly important aspects of clinical cancer biomarkers to-date to connect molecular innovation with clinical implementation to eventually improve patient care.

## Biomarkers in serum, plasma, urine and pleural fluid

### Diagnostic, prognostic and predictive: catching all in one test, and how early?

**Fig. 1:**
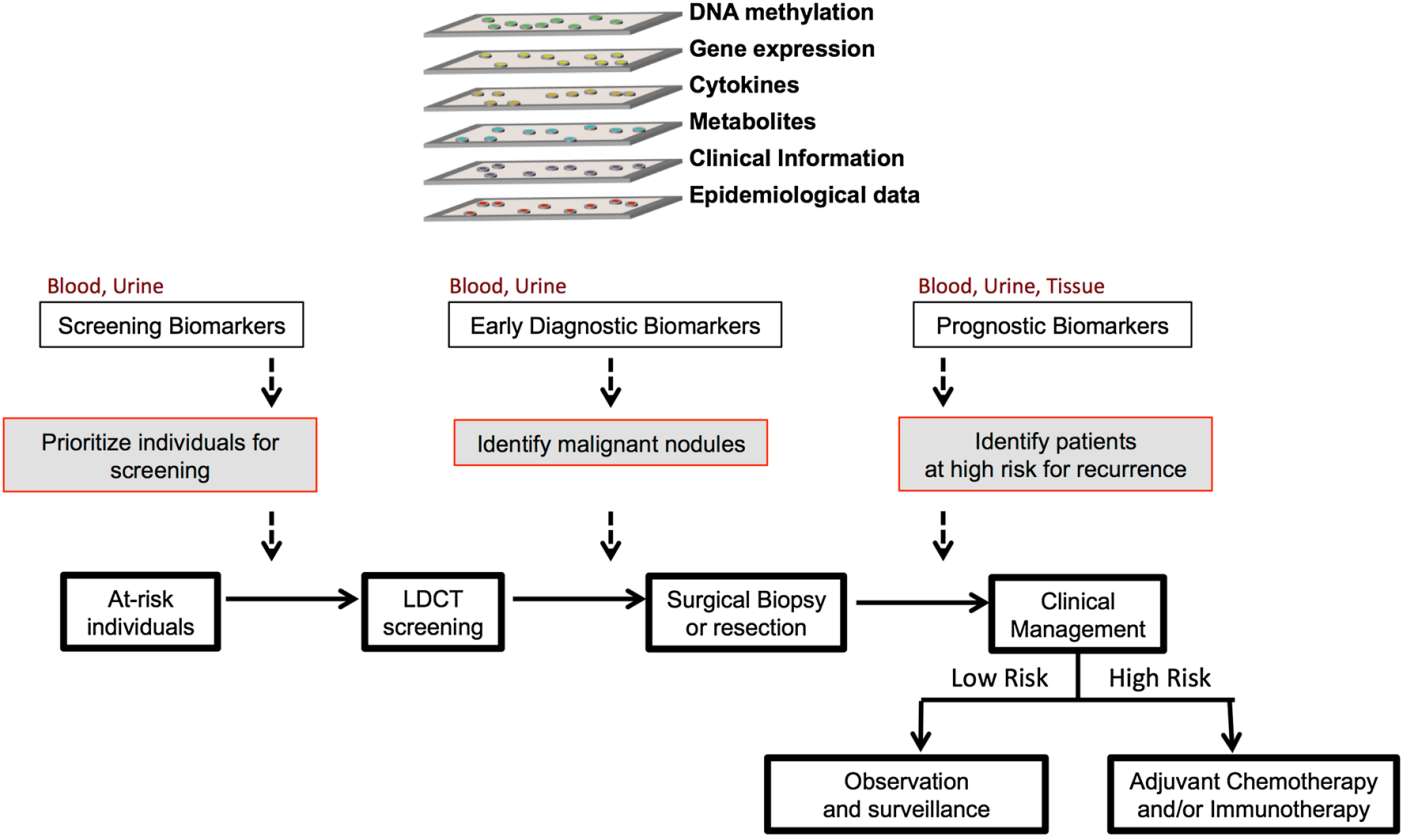
Biomarkers can help address the unmet needs of early stage lung cancer patients

More than half of all new lung cancer diagnoses are made in patients with locally advanced or metastatic disease, with few therapeutic options. However, screening efforts that use low-dose computed tomography (LDCT) are resulting in a greater proportion of lung cancers being diagnosed at an early, potentially curable, stage. Current guidelines for LDCT screening broadly include individuals based on age and history of heavy smoking. Biomarkers associated with lung cancer risk are needed to prioritize individuals for LDCT screening. LDCT detects a high number of nodules, of which fewer than 5 % are finally diagnosed as lung cancer. Thus, biomarkers are needed to help discriminate malignant nodules from benign or indolent lesions. In addition, prognostic biomarkers that molecularly categorize early stage patients after tumor resection would help identify those high risk for recurrence would benefit from adjuvant chemotherapy or innovative immunotherapy. In “Integration of multiple “omic” biomarkers: a precision medicine strategy for lung cancer” Ana I. Robles, NIH/National Cancer Institute, Bethesda, USA presented work on the identification of biomarkers in blood, urine, and resected tissues of early stage lung cancer patients. Elevated levels of pro-inflammatory cytokines IL-6, CRP and IL-8 were associated with lung cancer diagnosis. Significantly, IL-8 levels were elevated up to 5 years before diagnosis, suggesting its potential use as part of a screening program [3]. Moreover, a combined IL-6 and IL-8 prognostic classifier was associated with poor outcome in stage I lung cancer patients, including those with ≥30 pack-years of smoking (the relevant patient demographic targeted by LDCT screening) [4]. Using global and targeted metabolomics they uncovered a set of urine metabolites associated with lung cancer diagnosis, which has now been validated in a prospective study, where metabolite levels are elevated prior to clinically detectable disease [5, 6]. They also identified and validated prognostic biomarkers based on expression of genes and microRNA, and DNA promoter methylation in resected tissues [7–11]. Most recently, they integrated these biomarkers into a simple score that identified high-risk, therapy naive, stage I patients [11]. Current efforts are focused on translating these biomarkers into clinical tests that can help address the unmet medical needs of early stage lung cancer patients (Fig. 1).

Early diagnosis gets a new meaning when tumor-specific changes can be seen in the blood several years before clinical tumor is evident. Early pre-diagnostic tests may also change prognosis dramatically, as diagnosing lung cancer before reaching the size of 2 cm and before spreading to the lymph nodes can confer a 5-year survival of 80 %. In his talk “Serum microRNAs/enriched pathways in lung cancer 1–4 years before diagnosis—a pilot study from the HUNT Biobank, Norway”, Oluf D. Røe, Norwegian University of Science and Technology, Trondheim, Norway, presented the work of his group aiming at discovery of microRNAs, metabolites and proteins in serum samples 1–5 years before lung cancer diagnosis. Serum samples from the HUNT Biobank (https://www.ntnu.edu/hunt/about-hunt), Levanger, Norway were profiled with separate total microRNA sequencing (Illumina). The samples included lung adenocarcinoma (n = 4), squamous cell carcinoma (n = 5) and small-cell carcinoma (n = 5) cases collected 1–4 years before diagnosis, along with age and sex-matched non-cancer individuals (n = 28), ratio 1:2; the never smokers to ever-smokers ratio of controls was 50/50. The differentially expressed (DE) microRNAs were analyzed for enrichment by DIANA-miRPath v3. 0. The target genes of each microRNA included in the signature were determined with the DIANA-microT web server v5.0 and the union of all the microRNA targets was assessed for enrichment in KEGG pathways. Pathways with FDR-corrected *p* value <0.05 are reported in the results. The preliminary results detected 12 DE microRNAs in the adenocarcinomas plus squamous cell carcinomas versus controls as well as 9 DE microRNAs in small-cell carcinomas. Several pathways were enriched, including pathways for their respective cancer types (Table 1). They concluded that despite the small size of this pilot study, significantly DE microRNAs in serum of lung cancer patient were detected 1–4 years prior to diagnosis. These specific microRNAs also target cancer-specific pathways. These preliminary results are currently validated in a larger cohort, hopefully resulting in a clinical test in the near future.

**Table 1:** MicroRNAs in serum 1–4 years before diagnosis of lung cancer. Significantly differentially expressed microRNAs targeted genes of several pathways that were enriched, including known pathways of their respective cancer types

KEGG pathway	P value	#genes	#miRNAs
SCLC vs controls			
ECM-receptor interaction	7.85E-31	25	4
Small cell lung cancer	3.09E-06	24	6
NSCLC vs controls			
Dopaminergic synapse	8.82E-11	53	10
Non-small cell lung cancer	0.00142	17	10

How many years before a clinical diagnosis can a cancer signature be detected? In a large Norwegian study presented by Karina Standahl Olsen, Department of Community Medicine, UiT The Artic University of Norway, Tromsø, Norway the answer was “Blood gene expression profiles reflect temporality and clinical parameters up to 6 years before breast cancer diagnosis—The Norwegian Women and Cancer Post-genome cohort (Kvinner og Kreft studien). Since the understanding of time related aspects of systemic processes during carcinogenesis is very limited, this study aimed to identify time- and metastasis-related blood gene expression patterns present years before cancer diagnosis. Blood samples were collected prospectively from healthy, middle-aged women participating in the Norwegian Women and Cancer Post-genome cohort. Breast cancer cases were identified via linkage to the Cancer Registry of Norway, and matched controls were drawn from the cohort biobank. Full-blood gene expression was measured using Illumina Bead chips. The Cancer Registry provided information on time of diagnosis relative to screening visits, and on lymph node status. The included 441 case–control pairs were ranked according to the time interval between blood sampling and cancer diagnosis, providing information on blood gene expression up to six years before diagnosis. A non-parametric statistical method was developed to study changes in gene expression over time, named *curve group analysis*, which detects small gene expression differences that vary over time, and groups genes that display similar expression curves [12]. Blood gene expression differences between breast cancer cases and controls were dependent on time, and were strongest in the last year before diagnosis. Gene expression curves in the six years before diagnosis were only evident when stratifying cases according to mode of cancer detection and lymph node status. The study concludes that blood gene expression patterns do reflect clinical variability and temporality in the years before breast cancer diagnosis. These findings hold promise of increased insight into previously un-reachable aspects of systemic cancer biology. Blood gene expression patterns may be explored as potential biomarkers and/or used for development of tests [13].

**Fig. 2:**
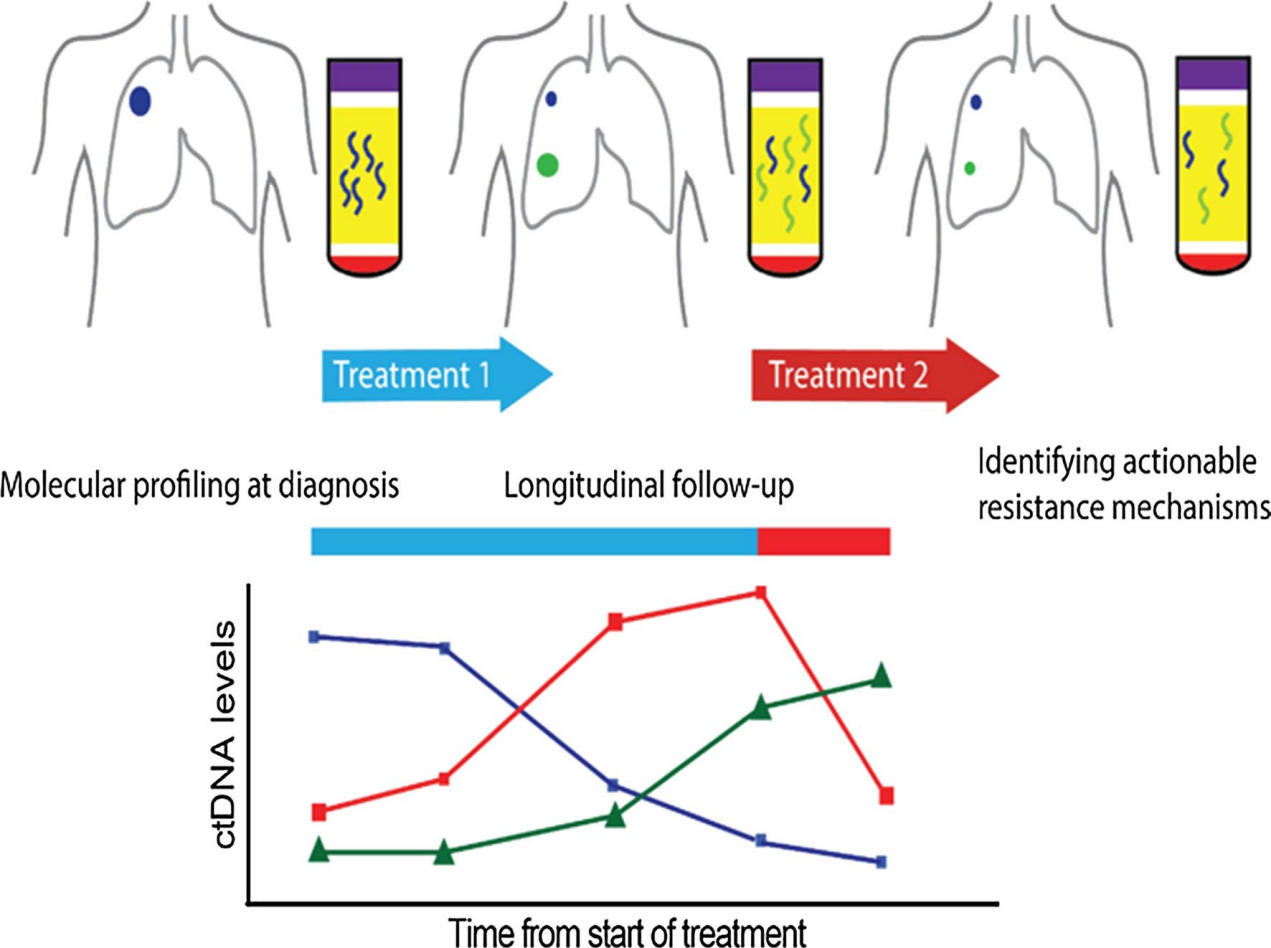
The potential of cell-free DNA for noninvasive cancer management Plasma cell-free circulating tumor DNA (ctDNA) analysis shows promises for noninvasive molecular stratification, monitoring treatment responses and identifying genetic mechanisms of resistance to guide optimal treatment strategies

In the presentation “The translational potential of circulating tumor DNA in oncology”, Dana W.Y. Tsui, from the Department of Pathology and Center for Molecular Oncology, Memorial Sloan Kettering Cancer Center, New York, USA, gave an overview of the field and talked about **t**he variety of clinical scenarios in which plasma DNA could be applied as a tool for noninvasive cancer management. This session outlined examples from different solid tumor, including breast, ovarian and lung cancers, to illustrate the application of circulating tumor DNA profiling for molecular stratification, monitoring tumor burden, identifying resistance mechanisms [14, 15]. The discussion also included an example from a research autopsy of a breast cancer patient to show that the analysis of circulating tumor DNA can reflect the dynamics of tumor clonal evolution from diagnosis to metastasis [15] The promise of circulating tumor DNA for detecting minimal residual disease, predicting prognosis and treatment response were illustrated in recent findings in breast and prostate cancers [16, 17]. Noninvasive plasma profiling shows great promise for precision medicine and is gradually being introduced to the clinical setting. Practical considerations for the clinical implementation of plasma DNA profiling, including issues with pre-analytical factors such as the effects of sample processing, as well as the requirement for clinical turn-around time, sample storage and informatics infrastructure, all need to be taken into consideration before effective clinical deployment (Fig. 2).

Decades of studies on circulating tumor cells (CTC) have shown promise, but still not found a place in the clinic, In his talk “Circulating Tumor Cells in early and recurrent breast cancer: Research and clinical applications” Vassilis Georgoulias, leading the research at the Department of Medical Oncology, School of Medicine, University of Crete, Greece reported data indicating that the detection of CTCs in patients with early stage breast cancer is an independent unfavorable prognostic factor for disease recurrence and disease-related death [18, 19] since CTCs cannot be eliminated by adjuvant chemotherapy or hormone treatment in almost 50 % of the patients, probably because of their non-proliferating and/or dormant state. In addition, the measurement of CTCs in patients with metastatic disease (MBC) revealed that their increased number is associated with a decreased overall survival whereas numerous studies have reported that their decrease after one cycle of chemotherapy is associated with improved survival. Immunofluorescence studies have demonstrated that CTCs express phosphorylated EGFR (pEGFR) as well as HER2 (+) in 60–70 % of EBC patients, irrespective of the HER2 status of the primary tumor cells.

In a proof of principle study, pre-treated patients with MBC and pEGFR-expressing CTCs were treated with gefitinib, a specific tyrosine kinase inhibitor of the EGFR. A median reduction of 96.4 and 94.1 % in CTC count was observed in 11 of the 17 patients after one treatment cycle; it is to note, that after the 3rd course, most detected CTCs were pEGFR (−). One patient achieved a partial response and in two patients the progression-free survival (PFS) was 16.0 and 19.0 months. Similar results were obtained with lapatinib, a dual EGFR and HER2 tyrosine kinase inhibitor, as well as with trastuzumab, a monoclonal antibody against HER2. A randomized phase II study evaluating trastuzumab versus observation in women with HER2 (−) early breast cancer and detectable CTCs before and after adjuvant chemotherapy demonstrated a significantly higher disease free survival (DFS) in the treated patients compared to those who received the standard treatment [19, 20]. These data support the phenotypic and biological characterization of CTCs providing valuable information regarding molecular targets that may lead to a more selected and individualized treatment of patients with breast cancer.

Malignant mesothelioma is an asbestos-induced, aggressive tumor with limited treatment options and very poor outcome; median survival is less than 12 months and 5-year survival rates of between 5 and 10 % have been reported [21]. Development of mesothelioma-specific biomarkers is an active area of research aimed not only at enhancing clinical care but also providing a means to screen at-risk asbestos exposed populations for early intervention [22]. Soluble mesothelin is the most intensively investigated mesothelioma biomarker and has been approved by the USA Food and Drug Administration as a tool for monitoring mesothelioma patient response and progression [23] The limited expression of the molecule on normal, nonmalignant tissue makes mesothelin an attractive therapeutic target as well as a diagnostic biomarker. Jenette Creaney, National Centre for Asbestos Related Disease, University of Western Australia presented “Mesothelin, discovery of a diagnostic marker that became a target”. For over fifteen years strategies have been pursued to target mesothelin-expressing cells using antibody-based therapies several of which are being evaluated in the clinical setting. Some of these studies have produced spectacular tumor regressions in some patients. Preliminary results of these early trials have been encouraging, although many different treatment strategies are being pursued ranging from more non-specific immunotherapy approaches to personalized neo-antigen vaccine development.

**Fig. 3:**
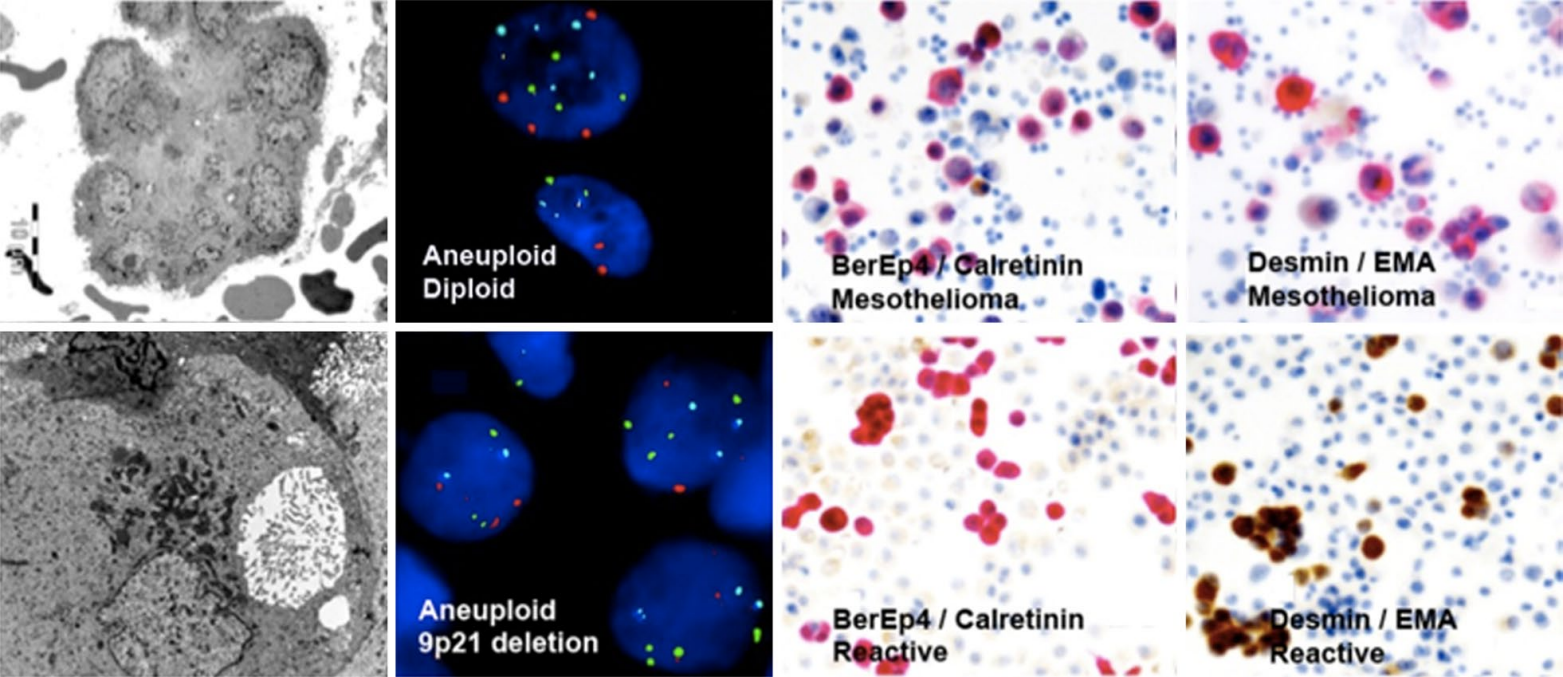
Early diagnosis of malignant mesothelioma in cell rich pleural effusions. Cytomorphology is combined with adjuvant analyses comprising electron microscopy (*left upper* and *left lower panels*), Fluorescence in Situ Hybridization (FISH) (*middle panels*) and dual immunocytochemistry (*right upper* and *right lower panels*); distinguishing malignant cells from reactive mesothelial cells and inflammatory cells (Courtesy of Katalin Dobra)

Pleural effusion is an underestimated and understudied biological matter that is minimal invasive and contains important disease-specific information. A conclusive diagnosis of both primary and metastatic tumours can actually be based on pleural effusion cytology and biomarker analysis of the cell-free fraction (Fig. 3) [24]. This could make an earlier diagnosis possible, which in turn may influence the effect of chemotherapy and patient survival.

The molecular landscape and bio signature of various malignancies is highly variable at this location and it requires detailed molecular characterisation [25–27] and optimized algorithms [28] to allow personalized treatment options and targeted therapy [29, 30]. In her presentation “Pleural effusion and bio-signature”, Katalin Dobra, Department of Laboratory Medicine, Division of Pathology Karolinska University Hospital in Huddinge, Sweden discussed their work.

Time to diagnosis by cytology versus histology in 77 epithelioid and mixed types of malignant pleural mesothelioma were studied. All diagnoses were supported by clinical findings, including CT scans. Clinical data, including evaluation of responses, were retrieved from hospital archives and survival data were obtained from the Swedish population database. The results showed that median time for diagnosis was 1 month less for cytology compared to histology. Preliminary data showed that the proportion of patients surviving 3 years was significantly better (p = 0,02) following a diagnosis based on effusion cytology, among treated patients 9/26 (38 %) versus only 1/23 (4 %), the median survival being 23 months and 14 months, respectively. The rate of initial responses to chemotherapy (stable disease + partial response) was slightly better in the cytology group. The earlier MM diagnosis obtained with effusion cytology seem to improve the overall survival after chemotherapy. Their findings show the importance of the cytological diagnosis and encourage the initiation of treatment as soon as the diagnosis is obtained.

## Biomarkers in tumors

### From the “gold-standard” immunohistochemistry to miR qISH, multi-level molecular analyses, immune checkpoint markers, DNA repair and novel clinical trial designs for diagnosis, stratification and avoiding overtreatment

Immunohistochemistry (IHC) has traditionally been employed in surgical pathology as an ancillary test in the analysis and classification of cancers. The availability of antibodies to cell specific proteins, and more recently to organ restricted transcription factors, has improved the accuracy of tumor diagnoses. Increasing numbers of tumors are defined by their underlying molecular alterations identifiable by IHC. Thus, as a protein based technique, IHC can act as a rapid and inexpensive surrogate for molecular studies. However, pre-analytical issues (e.g. improper tissue handling), analytical issues (e.g. less successful antibody clones, insufficient epitope retrieval, insensitive visualization systems) and post analytical issues (inconsistent reading and interpretation) frequently hamper the diagnostic utility of IHC.

In his talk “Immunohistochemistry in cancer diagnosis, the pitfalls and the future” Mogens Vyberg, Institute of Pathology, Aalborg University Hospital, Denmark explained how external quality assurance (EQA) of IHC, common definitions of controls, and digital image analysis are required to improve the reliability of IHC.

**Fig. 4:**
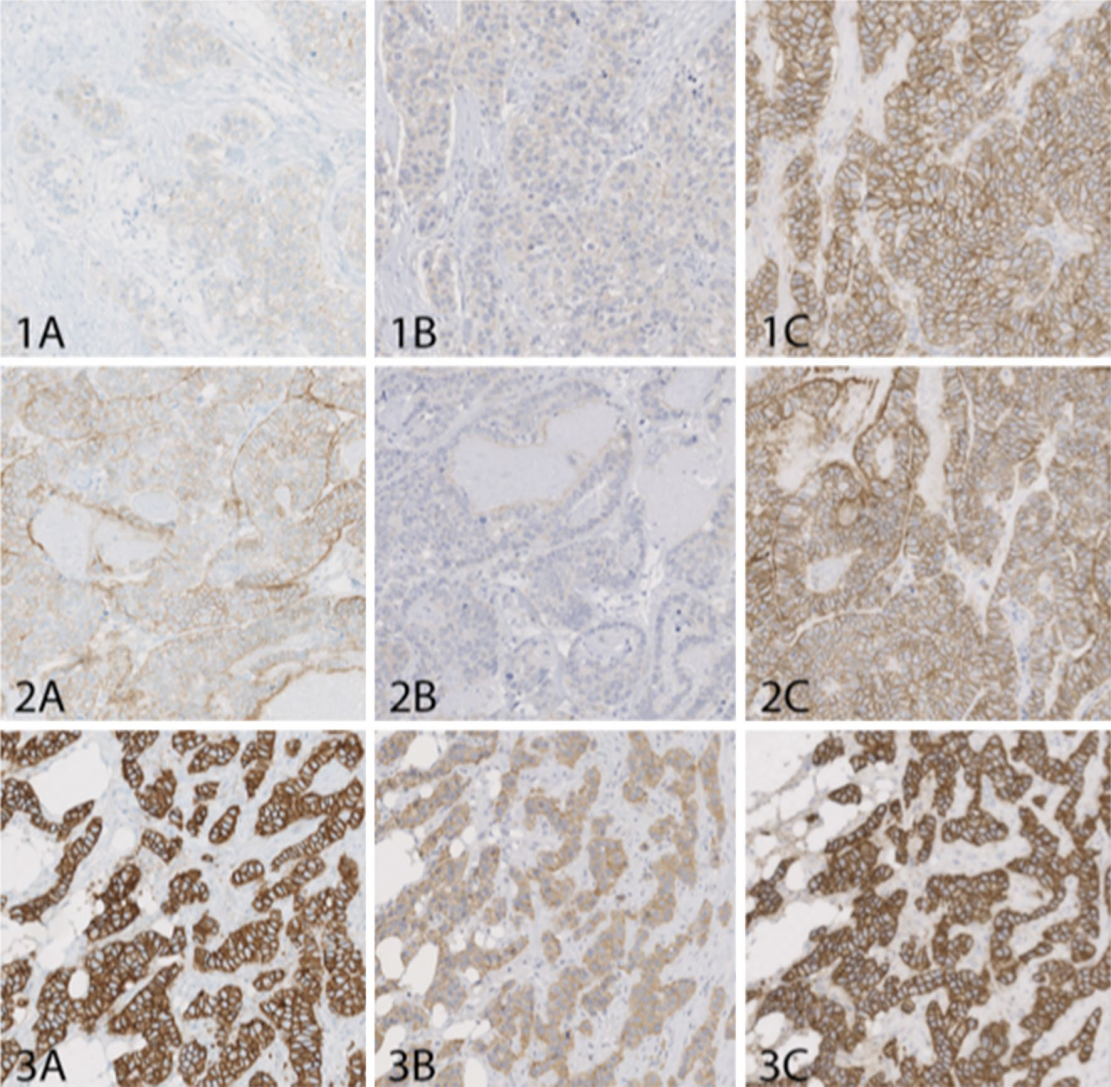
Poorly calibrated HER2 assays may give false negative and false positive results leading to erroneous patient treatment. Serial sections of a tissue micro array with cores from three breast ductal adenocarcinomas, marked* 1*,* 2* and* 3*, stained in three laboratories marked* A*,* B* and* C*. Core 1 (*upper row*): Carcinoma without HER2 gene amplification by FISH test and a 0/1+ immunostaining in labs* A* and* B*, while lab* C* obtains a 3+ staining. Lab C would not do a FISH test, and the patient would be offered an ineffective but costly and potentially hazardous HER2 targeted therapy. Core 2 (*middle row*): Carcinoma with a low but significant HER2 gene amplification obtained 2+ immunoreaction in lab* A*. Lab* B* obtained a 1+ staining which is false negative and the patient would not be offered HER2 targeted therapy in spite of the HER2 gene amplification. Lab* C *obtained a 3+ staining but in this case it would not influence the treatment. Core 3 (*lower row*): Carcinoma with high HER2 gene amplification and a 3+ immunoreaction obtained in lab* A* and* C*, while lab* B* obtained a 2+ staining. In a diagnostic setting this tumor would in lab* 2* be reflexed to FISH test for final HER2 status, increasing costs and turnaround time. The assay in lab* A* (reference lab) was based on an FDA approved kit, while the assays in lab* B* and* C* were laboratory developed (Courtesy of Mogens Vyberg)

More than 700 laboratories from about 85 countries are currently participating in the Nordic Immunohistochemical Quality Control (NordiQC) [31] EQA scheme. An expert board has assessed more than 30,000 IHC assays during 2003–2015. Overall, about 20 % of the staining results in the breast cancer IHC module and about 30 % in the general module have been deemed insufficient for diagnostic use. The causes of unsatisfactory results have been identified as mainly less successful antibodies (e.g., insensitive clones and poorly calibrated ready-to-use products) and suboptimal protocols (too dilute antibodies, erroneous epitope retrieval, less sensitive visualization systems and stainer platform issues). Individually tailored recommendations for protocol optimization, description of the best tissue controls to ensure appropriate calibration of the IHC assay and digital image analysis for HER2 stained breast cancers have generally improved the quality as well as inter-laboratory consistency of the IHC results. The increasing number of IHC assays for predictive markers makes continuous EQA mandatory. Figure 4 shows how poorly calibrated HER2 assays may give false negative and false positive results leading to erroneous patient treatment. Detailed description of the results of the NordiQC program is available on www.nordiqc.org.

Since the proposal of cancer immunosurveillance concept by Burnet and Smith more than a half-century ago, numerous attempts of cancer immunotherapy have been made. Currently, immune checkpoint blockade therapy is revolutionizing cancer therapy, where one target is PD-1, originally discovered by Dr. Honjo’s group at Kyoto University in 1992, a TCR-coinhibitory receptor and playing a crucial role in the checkpoint of T-cell self-tolerance. One of the discoverers of the PD1 antibody and its function, Nagahiro Minato, Department of Immunology and Cell Biology, Graduate School of Medicine, Kyoto University, Japan took us through “PD-1 checkpoint blockade for cancer immunotherapy: History and future perspectives”.

In 2002, his group reported that the PD-1 checkpoint also takes an important role in restraining endogenous anti-tumor immunity, and demonstrated that the blockade of the PD-1 checkpoint provides a potent therapeutic effect on tumors in animal models. The proposal for PD-1 checkpoint cancer immunotherapy was followed by a number of reports for beneficial effects of humanized anti-PD-1 or anti-PD-L1 antibodies in large-scale human clinical trials for various types of cancers since 2012. FDA approved humanized anti-PD-1 antibody for melanoma in 2014 and for non-small and small cell lung cancers in 2015, and currently hundreds of clinical studies including various combination therapies and many cancer types are underway worldwide. In parallel, studies on biomarkers affecting the efficacy of the therapy are also in progress from diverse aspects. PD-L1 expression can be induced on cancer cells in various conditions, including microenvironmental stress such as inflammation, and irreversible genomic changes in cancer cells. A recent report has indicated that varying proportions of multiple human cancers show recurrent structural changes in PD-L1 gene locus at 3′ UT region, which leads to a remarkable increase in PD-L1 expression [32]. Somatic mutations that occur frequently in cancer cells may lead to the emergence of potential neo-antigens, particularly when cancer cells have defective mismatch-repair capacity, recruiting and activating effector T cells with additional repertoire under checkpoint blockade [33]. Large-scale studies under the support of Society for Immunotherapy of Cancer, for instance, have reinforced a significant prognostic value of immunoscore with standardized methodology in multiple cancers [34]. Since the effects of checkpoint blockade immunotherapy count on endogenous immune response, verification of host immune status at tumor sites should also provide valuable biomarkers in his talk the history of PD-1 discovery, basic immuno biological studies, and several important future perspectives on PD-1 checkpoint blockade cancer immunotherapy were discussed.

Some cancers, including melanoma, kidney, and lung cancer, are naturally immunogenic, and can respond to checkpoint inhibitor therapy (anti-CTL4 or anti-PD1/L1). These agents block the physiologic stop signals that have been co-opted by tumor cells in order to evade a patient’s immune response. Checkpoint inhibitor blockade has demonstrated that a patient’s immune system can eliminate even widely metastatic cancer. There has been attention on defining the prognostic and predictive power of checkpoint inhibitors. The PD-L1 protein has been explored for its prognostic and predictive ability, discussed by Robert A. Anders, Johns Hopkins University, Baltimore, USA, in his talk “Prognostic and predictive role of PD1 and PD-L1 in cancers”. The prognostic role of PD-L1 expression in cancers is tumor type dependent. On the one hand PD-L1 expression indicates a better prognosis in melanoma and lung cancer. On the other hand it is a poor prognostic in gastric and kidney cancer. There are both technical and biologic reasons for these discrepancies. First, determining if a cancer expresses PD-L1 is complicated by the required level of expression, the type of cells expressing PD-L1 and the cellular location of PD-L1.

Second, each cancer type can have different tumor microenvironments.

**Fig. 5:**
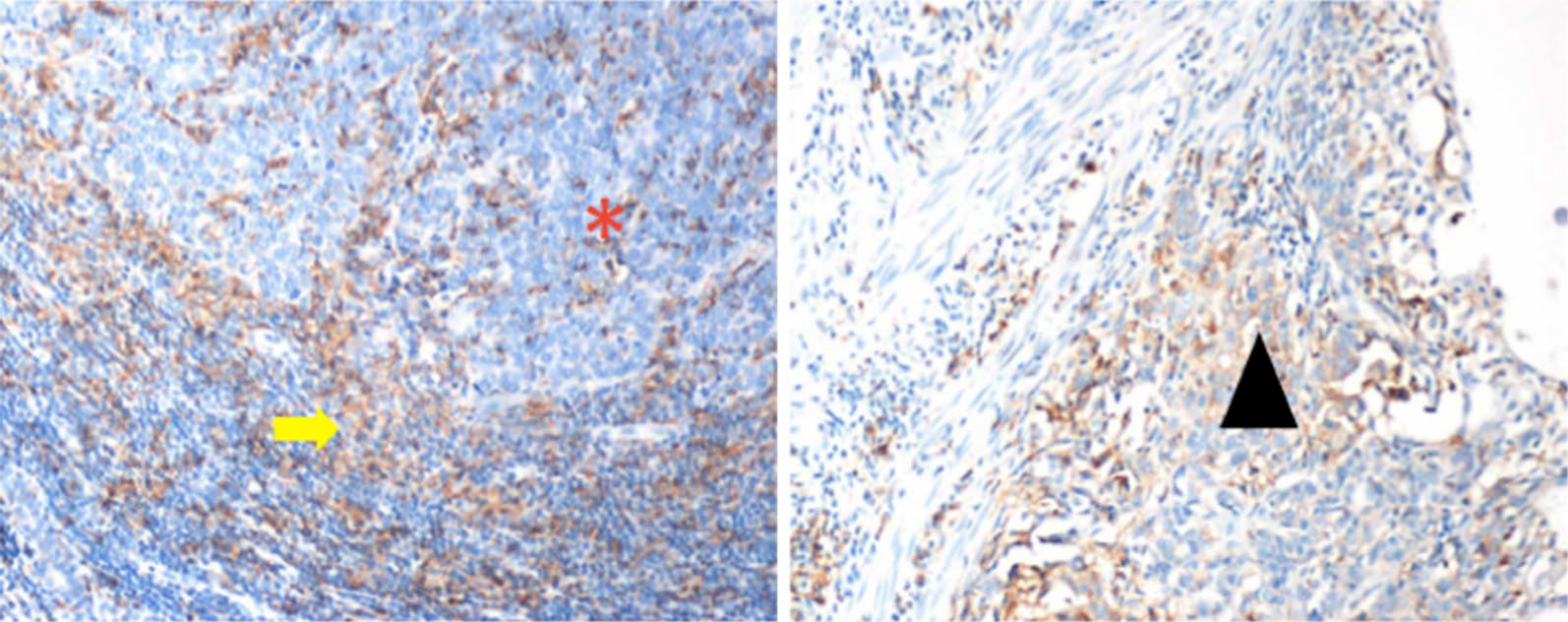
Expression of PD-L1 in gastric adenocarcinoma. There is expression of PD-L1 as detected by immunohistochemical staining (SP142) in the tumor infiltrating immune cells (*yellow arrow*) and not on the malignant adenocarcinoma cells (red *). Cell surface expression of PD-L1 is evident on adenocarcinoma cells (*black arrow head*) in another PD-L1 stained sample (Courtesy of Robert Anders)

The predictive value of PD-L1 expression is becoming clearer. An early anti-PD-1 trial showed PD-L1 expression had a ~40 % positive predictive value and a 100 % negative predictive value in therapeutic response to PD-1 blockade [35]. Subsequent studies mirrored that trend but were not so absolute. Now PD-L1 expressing cancers (not broken down by tumor type) have a ~45 % chance of responding to therapy compared to ~15 % for PD-L1 negative cancers [36]. PD-L1 expression on a cancer indicates a patient is more likely to respond to anti-PD-L1 blockade but should not be the single criterion for receiving therapy. Not every tumor immune microenvironment is identical and they are not all dependent upon PD-L1 expression (Fig. 5). Taken together, PD-L1 is not a perfect biomarker for response to anti-PD1/PD-L1 therapy. Increasingly the integration of the type, location and function of tumor associated immune cells are important in determining patient response. Furthermore genomic data has shown that tumor cell mutational load is another key component of the immune response and a biomarker of response to immune based cancer therapy [37–40]. In particular genomic data from the cancer genome atlas (TCGA) was shown to be an independent predictive biomarker in response to checkpoint inhibition [41].

The role of complex cancer biomarkers was further discussed by Anne-Lise Børresen-Dale, Department of Cancer Genetics, Institute for Cancer Research, Oslo University Hospital The Norwegian Radiumhospital, Oslo, Norway in her talk “Role of multilevel molecular analyses in reducing over-treatment in breast cancer”. Several known prognostic factors are used to identify breast cancer patients with an unfavorable prognosis, such as tumor size, histological grade, hormone receptor status and axillary lymph node metastasis. However, a large majority of early stage breast cancer patients with small primary tumors receive such treatment without being at risk of developing recurrent disease. Thus a more precise stratification for treatment decisions is thus highly needed. A detailed characterization of the individual breast tumors at the molecular level may improve individualized prognostication and treatment decisions. High-throughput molecular analyses of tumor tissue at DNA, (copy number and methylation) mRNA, miRNA, protein (using reverse phase protein arrays, RPPA), and metabolic (HR-MAS MR) levels were performed. Dr. Børresen-Dale’s group has explored to which extent combining the various profiles derived from each level, can further subdivide the initially discovered PAM 50 expression subclasses [42, 43] and improve prognostic potential in individual patients. Combined analyses of gene regulation at various levels may point to specific biological functions and molecular pathways that are deregulated in individual breast cancers and may reveal novel subgroups of patients for tailored therapy and monitoring [44].

Several of the molecular layers, both individually and combined, could split the ER positive Luminal A group, the most frequent subtype of breast cancer, into two subgroups with different survival. The identification of patients with low-risk Luminal A tumors using a set of methylation markers [45] or a set of miRNAs [46] will guide in selecting patients not benefitting from further adjuvant treatment.

Gastric cancer is the fourth most common cancer and the second leading cause of cancer-related death worldwide. Chemotherapy both in resectable and advanced disease has only limited efficacy. There are no clinically validated prognostic or predictive biomarkers, and in his presentation “JWA and XRCC1 as predictive and prognostic factors in gastric cancer”, Jianwei Zhou, Department of Molecular Cell Biology & Toxicology, Cancer Center, School of Public Health, Nanjing Medical University, People’s Republic of China, took us through the journey of discovering the JWA and how it could be a valid clinical marker alone and together with XRCC1.

JWA and XRCC1 expression status in resectable gastric cancer cases treated with adjuvant chemotherapy compared with surgery was tested in a first training cohort, a second testing cohort and finally in a validation cohort (n = 80, 374 and 385 respectively) [47] showing that protein levels of both were significantly downregulated in gastric cancer lesions compared with adjacent non-cancerous tissues. Low tumoral JWA or XRCC1 expression significantly correlated with shorter overall survival (OS) and multivariate regression analysis showed that low JWA and XRCC1 expression, separately and together, were independent negative markers of OS. Adjuvant fluorouracil-leucovorin-oxaliplatin (FLO) significantly improved OS compared with surgery alone. However, this effect was evident only in the JWA or XRCC1 low expression group; similar effect was also observed in patients with fluorouracil- leucovorin-platinum (FLP) regimen for JWA and XRCC1. Therefore, JWA and XRCC1 protein expression in tumor are novel candidate prognostic markers and predictive factors for benefit of adjuvant platinum-based chemotherapy (FLO or FLP) in resectable human gastric carcinoma.

Further molecular analyses on the roles of JWA and XRCC1 were performed in cisplatin sensitive (SGC7901, BGC823) and resistant (SGC7901/DDP, BGC823/DDP) human gastric cancer cells and unraveled mechanistically that JWA regulated cisplatin induced DNA damage and apoptosis through CK2—P-XRCC1—XRCC1 pathway, indicating a putative target for reversing cisplatin resistance in gastric cancer [48].

The first two microRNAs (miRNAs) discovered—lin-4 in 1993 and let-7 in 2000—have critical roles in worm development, as loss of either results in retarded worm development due to lack of cell differentiation. Consequently, when miRNAs were recognized as a large and highly conserved class of genes in 2001, scientists quickly surmised miRNAs potential in cancer biology. Indeed, cancers are characterized by altered miRNA expression profiles and individual miRNAs can act as oncogenes and tumor suppressors. In his presentation “From worm to man: Discovery of microRNA and current potential as clinical biomarkers and targets”, Pål Sætrom, Department of Computer and Information Science took us through why microRNAs are considered promising candidates both as cancer biomarkers and as therapeutic targets. To illustrate, PubMed has presently indexed more than 60,000 papers and abstracts on miRNAs; 40 % of these are about cancer.

MicroRNAs are short, non-coding RNAs comprising 18–23 nucleotides, and more than 2000 microRNAs have been reported in the human genome. MicroRNAs exert cell-specific activity by binding to the 3′UTR of mRNAs and thereby negatively regulating translation. The mature microRNAs are bound in protein complexes in which they are stable in tissues and blood. Most current therapeutic strategies aim to address tumor imbalance in miRNA expression and individual miRNA’s role as oncogene or tumor suppressor. Specifically, tumors with down-regulated miRNAs that are tumor suppressors are treated by introducing molecules that mimic miRNAs, whereas tumors with up-regulated oncogenic miRNAs are treated by introducing molecules (anti-miRs) that prevent the oncomirs from binding their target RNAs. Currently, a mimic for miR-34 is in clinical trials for liver cancer, whereas multiple anti-miRs are at the preclinical stage for different cancers [49].

Most current therapeutic strategies aim to affect miRNAs canonical roles in regulating protein coding genes post transcription in the cytoplasm. However, miRNAs can also regulate genes by affecting gene transcription in the nucleus. Others and we have shown that artificial miRNA-like RNAs, so-called short activating RNAs (saRNAs) can transcriptionally up-regulate target genes [50]. One such saRNA, targeting the transcription factor CCAAT/enhancer-binding protein alpha (CEBPA), can reduce tumor burden and improve liver function in liver cancer models and is currently in clinical Phase 1.

**Fig. 6:**
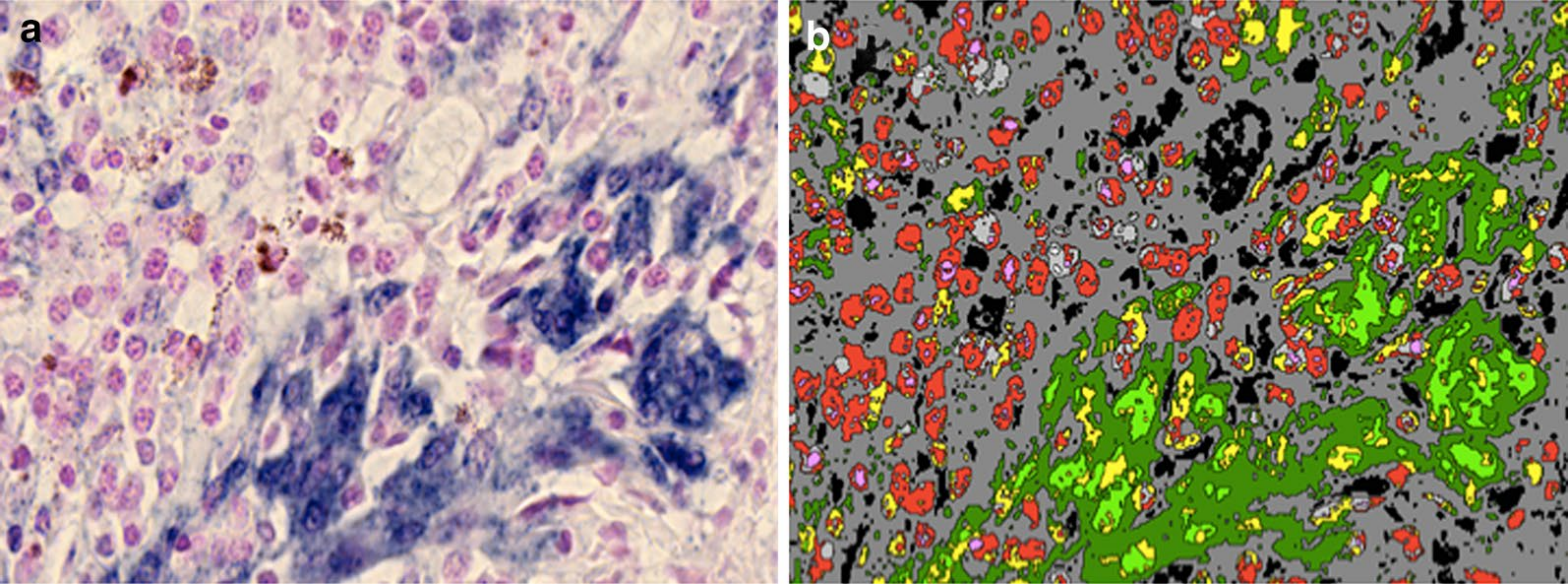
MiR-21 qISH slide processing. Sample image acquired from a digital whole slide (**a**) and color segmented image after pixel classification (**b**). The classification was based on the various colors identified in the stained section, e.g. the intense *blue color* in A (miR-21 ISH staining) is translated into *bright green* in **b**. Total area calculations and relative area fractions can be obtained from the color segmented image For further details, see Eriksen et al. 2016 [46] (Courtesy of Boye Schnack Nielsen)

Tissue slide-based assays provide qualitative (tumor compartment) and semi-quantitative (expression levels) of miRNA expression at single-cell resolution in clinical tumor specimens and show a potential prognostic value in urothelial carcinoma, colon, lung, breast cancer, melanoma and glioma [51]. However, imaging analysis of microRNA expression is not in clinical use so far. MicroRNA-21 (miR-21) is the most prevalent microRNA in many tumors and regulates expression of tumor suppressors like PTEN and PDCD4. In “miR-21 quantitative in situ hybridization and its value as biomarker in a clinical setting”, Boye Schnack Nielsen, Molecular Histology, Bioneer A/S, Hørsholm, Denmark, presented data related to the performance of the miR-21 in situ hybridization (ISH) using locked nucleic acid (LNA) probe technology [52] based on digital whole slides. miR-21 is expressed at increased levels in tumor tissue compared to normal tissue and is positively associated with poor prognosis, e.g. in stage II colon cancer [53]. Image analysis performed on digital whole slides showed increased precision compared to previous methods (Fig. 6). The miR-21 was primarily located in stromal fibroblast-like cells [54]. Moreover, miR-21 qISH expression levels were independent of tumor heterogeneity [55], which is a prerequisite for robust cancer biomarkers. The study concluded that the miR-21 qISH expression estimates, together with visual examination of the staining patterns, may prove useful for better stratification of the cancer patients, and that the miR-21 qISH approach may work as a model system for microRNA qISH analyses in general.

MicroRNAs can function as predictors and prognosticators in several types of cancer. For malignant pleural mesothelioma (MPM) prognosis is poor, but a subset of patients treated with multimodal treatment can have a long survival. However, this treatment is highly invasive and stressful for the patients, and there are no markers to predict outcome in this patient group. In “MicroRNAs as prognostic biomarkers for survival in surgically treated malignant pleural mesothelioma patients”, Michaela B Kirschner, Division of Thoracic Surgery, University Hospital Zurich, Switzerland presented the possibility of using a microRNA expression signature as prognostic factor for patients considered for multimodality treatment including surgical resection. This study used three independent series of MPM tumor samples: Series 1–3 were samples from 48 extrapleural pneumonectomies (EPP), 43 pleurectomy/decortications (P/D) and 100 EPP with matching diagnostic biopsies respectively. MicroRNA expression was analyzed by RT-qPCR and associations with survival are assessed by Kaplan–Meier log-rank analysis. In addition, a microRNA expression signature (miR-Score) for prediction of good prognosis (≥20 months survival) was built using binary logistic regression modeling, and evaluated by receiver operating characteristics curve analysis. The miR-Score including 6 microRNAs (miR−21, −23a, −30e, −221, −222, −31) was able to predict a good prognosis with an accuracy of 92.3 % in patients undergoing EPP and an accuracy of 71.9 % in patients receiving P/D. Score-positive patients showed increased median overall survival of 23 and 9 months for EPP and P/D, respectively. Hazard ratios for score-negative patients were 4.12 (95 % CI: 2.03–8.37, p = 0.00001) for EPP and 1.93 (95 % CI: 1.01–3.69, p = 0.047) for P/D. Furthermore, adding the miR-Score to a prognostic model consisting of clinical factors resulted in improved accuracy [56].

Further investigations on the effect of chemotherapy and the prognostic value of the miR-Score in series 3 are currently ongoing. In conclusion this study has identified a novel microRNA signature with prognostic value in MPM patients. Ongoing validation and refinement of the miR-Score has the potential to provide a novel biomarker for more accurate selection of MPM patients considered for multimodality treatment.

DNA repair is essential for cell survival and prevention of mutations and cancer. In addition, DNA repair is intimately integrated with immunity. In “DNA Repair in diagnosis and therapy of cancer—opportunities and problems”, Hans E. Krokan, Department of Cancer Research and Molecular Medicine, Norwegian University of Science and Technology (NTNU) Trondheim, Norway, presented a historical overview of the DNA repair field an current implications. It has been known since 1968 that DNA excision repair deficiency causes sensitivity to UV-light and skin cancer in *Xeroderma pigmentosum* [57]. It is now known that more common cancers can be caused by unrepaired DNA damage and repair deficiencies. Thus, inactivation of double strand break repair (DSBR) due to BRCA1 or 2 gene mutations results in defective DSBR and strongly increased risk of early onset breast cancer and ovarian cancer. A potential upside is that BRCA1/2-defective cancers are sensitive to single strand break repair protein poly(ADP)ribose polymerase (PARP) inhibitors, due to synthetic lethality [58]. DNA mismatch-repair deficiency similarly causes hereditary non-polyposis colorectal cancer and several less common other cancers, the diagnosis of which is important to initiate preventive measures. Glioblastoma is a deadly form of brain tumor that is frequently deficient in direct repair of O^6^ methyl guanine by O^6^-meG-DNA methyltransferase (MGMT). MGMT-deficient tumors are sensitive to the methylating agent temozolomide (TMZ), whereas MGMT-overexpression causes TMZ resistance. Furthermore, untargeted DNA-cytosine deamination by AID/APOBECs causes increased genomic uracil and AID/APOBEC mutational signatures in B-cell malignancies and several other types of cancer. In line with this, it was observed that clustered mutations (*kataegis)* in B-cell malignancies predominantly carry AID-hotspot mutational signatures [59]. Thus, AID/APOBEC-induced mutagenic U: G mismatches in DNA left unrepaired may be one fundamental and relatively common cause of several malignancies.

With the availability of widespread genomic sequencing, and the introduction of specific targeted agents for subsets of patients with adenocarcinoma, survival for patients with non-squamous metastatic non-small cell lung cancer (NSCLC) squamous cell cancer (SCC) has significantly improved [60, 61]. Comprehensive genomic surveys, have also vastly improved our understanding of the mutational profile of SCC [62]. The Cancer Genome Atlas Project (TCGA) extensively profiled 178 SCC tumor specimens for genomic alterations and identified that TP53 was almost universally mutated in the tumor samples, but other genes such as CDKN2A/RB1, NFE2L2/KEAP1/CUL3, PI3 K/AKT, and SOX2/TP63/NOTCH1 signaling pathways were also commonly altered [62].

**Fig. 7:**
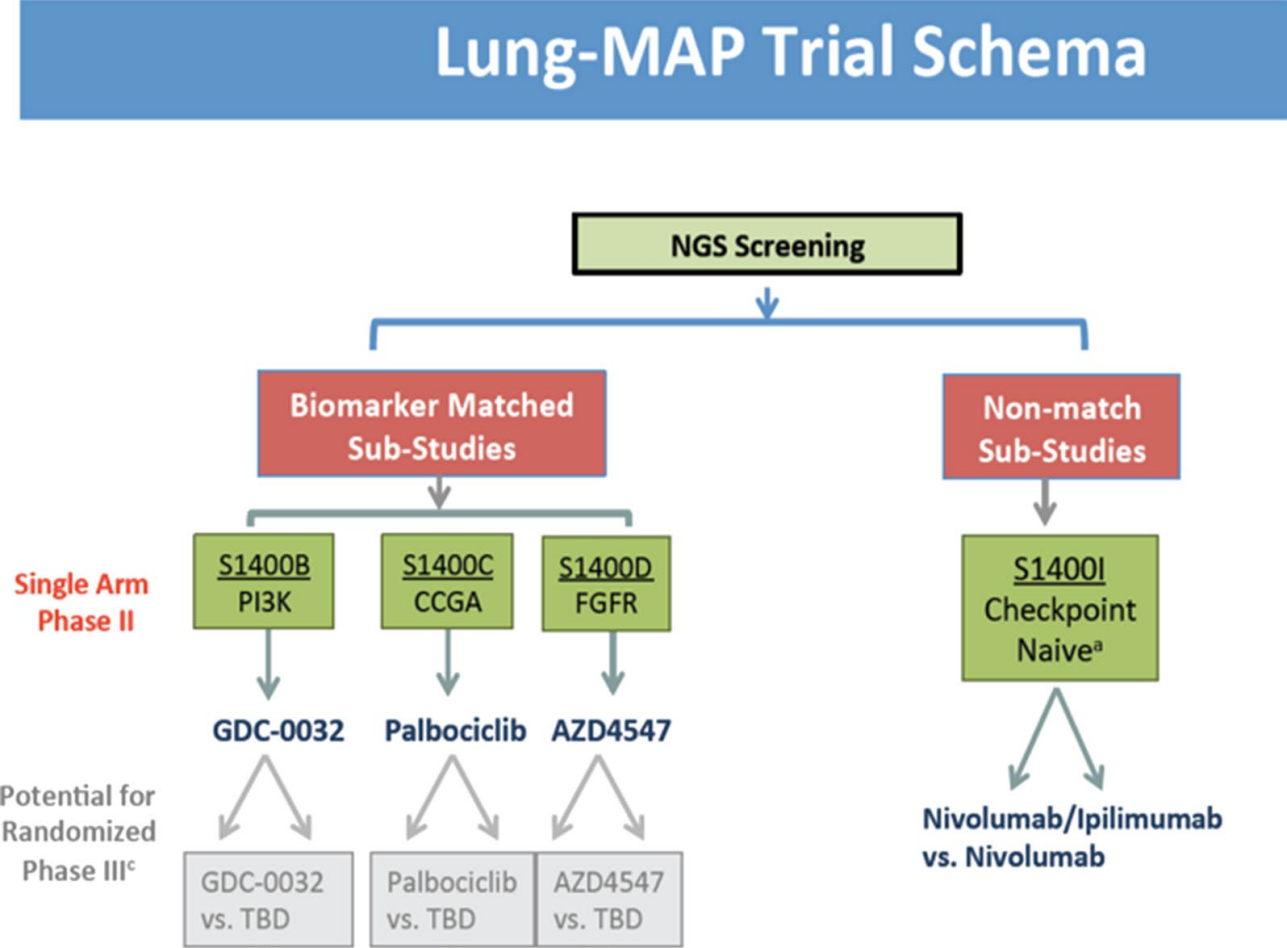
Trial Schema of the Lung-MAP study

Due to the genetic diversity and lack of clear oncogenic drivers in this disease, there is a need to develop clinical trials solely focused on SCC that can evaluate single agent and combination targeted therapies. In “Novel Clinical Trial design including biomarkers: The LUNG-MAP (Lung Master protocol, S1400) study”, Vassiliki Papadimitrakopoulou, Department of Thoracic/Head and Neck Medical Oncology, MD Anderson, Houston, USA, described the Lung-MAP (Master Protocol), an umbrella master multi study protocol that incorporates genomic testing of tumors through a next generation sequencing (NGS) platform (Foundation Medicine) for patients with SCC after progression on first line therapy (Fig. 7). This protocol represents partnership engaging the National Cancer Institute (NCI) and its Thoracic Malignancies Steering Committee (TMSC), the Foundation of the NIH (FNIH), the pharmaceutical industry, advocacy groups such as Friends of Cancer Research (FOCR), and the Federal Drug Administration (FDA). The main goal is to identify safe and effective regimens that match predictive biomarkers with targeted drugs. After genomic testing, patients are randomized into one of several sub-studies, each comparing an experimental targeted therapy with standard of care therapy, based on identification of candidate predictive biomarkers associated with each sub-study. New trials addressing alternative targets are being planned and each sub-study opens and closes independently of others. The study offers patients both targeted (matched sub-studies) and immunotherapy treatments (non-match), all within the umbrella master protocol design (schema).

## Handling of multiple datasets for biomarker discovery and validation

### Bioinformatics is one of the most important keys in transforming molecular data to a clinically valid test

Clinical, epidemiological, environmental, molecular, and genetic data related to cancer are becoming more and more available. A main computational analysis task of these data is to identify the minimal-size sets of biomarkers and biosignatures that collectively carry all the information for optimal prediction or diagnosis of the outcome of interest (e.g., cancer stage, survival, metastasis, etc.). In his talk “Automated Computational Discovery of Biomarkers and Biosignatures from Data Using Machine Learning” Ioannis Tsamardinos, Department of Computer Science, University of Crete, Heraklion, Greece takes on his experience on biosignatures with these properties which provide useful insight to the causal mechanisms of disease or can be used for devising diagnostic tests of minimal cost. Computational methods that try to solve this problem may for example return a minimum set of genes and environmental factors whose interactions, collectively best predict metastasis. Tsamardinos also presented the computational problems arising when trying to identify biosignatures, such as identifying one or all equivalent biosignatures, constructing predictive models from their measurements, estimating the performance of the predictions, tuning the algorithms for optimal prediction performance, identifying anomalous cases (e.g., outliers), and estimating the information value of obtaining new samples. Finally he presented related computational tools, libraries, and an automated, intelligent analysis pipeline for use by a non-expert that can perform a quality-analysis with a few clicks. The pipeline is demonstrated on several cancer prediction and diagnostic related problems.

## Conclusions

Through these Symposium presentations, the plurality, the various applications but also the pitfalls of current and potential future cancer biomarkers have been demonstrated. The use of tumor tissue is still the gold standard for diagnostic, prognostic and predictive biomarkers, including immunoscore of TILs, but circulating cells, tumor DNA and microRNA in blood are currently entering clinical practice. Multi-omics, poly-markers or multi-level tests may also be of high importance, as single type of molecules rarely can be sufficient to describe heterogeneous tumors. Pleural fluid and urine contain clinically relevant biological information, exemplified in mesothelioma and lung cancer. Bioinformatics is key to analysis, discovery and validation of biomarkers. Clinical use of biomarkers is hampered by their variable sensitivity and/specificity due to technical/laboratory variations and the heterogeneity of cancer phenotypes. Analysis of both ready-to-use open source high-throughput data as the TCGA but also of large prospective biobanks as the HUNT will be crucial for the timely discovery of non-invasive early diagnostic as well as predictive and prognostic markers. Finally, the integration of molecular knowledge in clinical trials was highlighted and seems likely be a key point for every future clinical study.

## References

[1] Buchbinder EI, Desai A (2016). CTLA-4 and PD-1 pathways: similarities, differences, and implications of their inhibition. Am J Clin Oncol.

[2] Lowy DR, Collins FS (2016). Aiming high-changing the trajectory for cancer. N Engl J Med.

[3] Pine SR, Mechanic LE, Enewold L, Chaturvedi AK, Katki HA (2011). Increased levels of circulating interleukin 6, interleukin 8, C-reactive protein, and risk of lung cancer. J Natl Cancer Inst.

[4] Ryan BM, Pine SR, Chaturvedi AK, Caporaso N, Harris CC (2014). A combined prognostic serum interleukin-8 and interleukin-6 classifier for stage 1 lung cancer in the prostate, lung, colorectal, and ovarian cancer screening trial. J Thorac Oncol.

[5] Mathe EA, Patterson AD, Haznadar M, Manna SK, Krausz KW (2014). Noninvasive urinary metabolomic profiling identifies diagnostic and prognostic markers in lung cancer. Cancer Res.

[6] Haznadar M, Cai Q, Krausz KW, Bowman ED, Margono E (2016). Urinary metabolite risk biomarkers of lung cancer: a prospective cohort study. Cancer Epidemiol Biomark Prev.

[7] Yanaihara N, Caplen N, Bowman E, Seike M, Kumamoto K (2006). Unique microRNA molecular profiles in lung cancer diagnosis and prognosis. Cancer Cell.

[8] Saito M, Schetter AJ, Mollerup S, Kohno T, Skaug V (2011). The association of microRNA expression with prognosis and progression in early-stage, non-small cell lung adenocarcinoma: a retrospective analysis of three cohorts. Clin Cancer Res.

[9] Akagi I, Okayama H, Schetter AJ, Robles AI, Kohno T (2013). Combination of protein coding and noncoding gene expression as a robust prognostic classifier in stage I lung adenocarcinoma. Cancer Res.

[10] Okayama H, Schetter AJ, Ishigame T, Robles AI, Kohno T (2014). The expression of four genes as a prognostic classifier for stage I lung adenocarcinoma in 12 independent cohorts. Cancer Epidemiol Biomark Prev.

[11] Robles AI, Arai E, Mathe EA, Okayama H, Schetter AJ (2015). An integrated prognostic classifier for stage i lung adenocarcinoma based on mRNA, microRNA, and DNA methylation biomarkers. J Thorac Oncol.

[12] Lund E, Holden L, Bovelstad H, Plancade S, Mode N (2016). A new statistical method for curve group analysis of longitudinal gene expression data illustrated for breast cancer in the NOWAC postgenome cohort as a proof of principle. BMC Med Res Methodol.

[13] Pantel K, Diaz LA, Polyak K (2013). Tracking tumor resistance using ‘liquid biopsies’. Nat Med.

[14] Dawson SJ, Tsui DW, Murtaza M, Biggs H, Rueda OM (2013). Analysis of circulating tumor DNA to monitor metastatic breast cancer. N Engl J Med.

[15] Murtaza M, Dawson SJ, Tsui DW, Gale D, Forshew T (2013). Non-invasive analysis of acquired resistance to cancer therapy by sequencing of plasma DNA. Nature.

[16] Garcia-Murillas I, Schiavon G, Weigelt B, Ng C, Hrebien S (2015). Mutation tracking in circulating tumor DNA predicts relapse in early breast cancer. Sci Transl Med..

[17] Romanel A, Gasi Tandefelt D, Conteduca V, Jayaram A, Casiraghi N (2015). Plasma AR and abiraterone-resistant prostate cancer. Sci Transl Med..

[18] Braun S, Pantel K, Muller P, Janni W, Hepp F (2000). Cytokeratin-positive cells in the bone marrow and survival of patients with stage I, II, or III breast cancer. N Engl J Med.

[19] Xenidis N, Perraki M, Kafousi M, Apostolaki S, Bolonaki I (2006). Predictive and prognostic value of peripheral blood cytokeratin-19 mRNA-positive cells detected by real-time polymerase chain reaction in node-negative breast cancer patients. J Clin Oncol.

[20] Georgoulias V, Bozionelou V, Agelaki S, Perraki M, Apostolaki S (2012). Trastuzumab decreases the incidence of clinical relapses in patients with early breast cancer presenting chemotherapy-resistant CK-19m RNA-positive circulating tumor cells: results of a randomized phase II study. Ann Oncol.

[21] Roe OD, Stella GM (2015). Malignant pleural mesothelioma: history, controversy and future of a manmade epidemic. Eur Respir Rev.

[22] Panou V, Vyberg M, Weinreich UM, Meristoudis C, Falkmer UG (2015). The established and future biomarkers of malignant pleural mesothelioma. Cancer Treat Rev.

[23] Creaney J, Dick IM, Robinson BW (2015). Discovery of new biomarkers for malignant mesothelioma. Curr Pulmonol Rep.

[24] Hjerpe A, Ascoli V, Bedrossian CW, Boon ME, Creaney J (2015). Guidelines for the cytopathologic diagnosis of epithelioid and mixed-type malignant mesothelioma. Complementary statement from the international mesothelioma interest group, also endorsed by the international academy of cytology and the papanicolaou society of cytopathology. Acta Cytol.

[25] Sun X, Dobra K, Bjornstedt M, Hjerpe A (2000). Upregulation of 9 genes, including that for thioredoxin, during epithelial differentiation of mesothelioma cells. Differentiation.

[26] Sun X, Wei L, Liden J, Hui G, Dahlman-Wright K (2005). Molecular characterization of tumour heterogeneity and malignant mesothelioma cell differentiation by gene profiling. J Pathol.

[27] Mundt F, Johansson HJ, Forshed J, Arslan S, Metintas M (2014). Proteome screening of pleural effusions identifies galectin 1 as a diagnostic biomarker and highlights several prognostic biomarkers for malignant mesothelioma. Mol Cell Proteom.

[28] Mundt F, Nilsonne G, Arslan S, Csuros K, Hillerdal G (2013). Hyaluronan and N-ERC/mesothelin as key biomarkers in a specific two-step model to predict pleural malignant mesothelioma. PLoS ONE.

[29] Sun X, Gulyas M, Hjerpe A, Dobra K (2006). Proteasome inhibitor PSI induces apoptosis in human mesothelioma cells. Cancer Lett.

[30] Szulkin A, Nilsonne G, Mundt F, Wasik AM, Souri P (2013). Variation in drug sensitivity of malignant mesothelioma cell lines with substantial effects of selenite and bortezomib, highlights need for individualized therapy. PLoS ONE.

[31] Vyberg M, Nielsen S (2016). Proficiency testing in immunohistochemistry–experiences from Nordic Immunohistochemical Quality Control (NordiQC). Virchows Arch.

[32] Kataoka K, Shiraishi Y, Takeda Y, Sakata S, Matsumoto M (2016). Aberrant PD-L1 expression through 3′-UTR disruption in multiple cancers. Nature.

[33] Stronen E, Toebes M, Kelderman S, van Buuren MM, Yang W (2016). Targeting of cancer neoantigens with donor-derived T cell receptor repertoires. Science.

[34] Galon J, Fox BA, Bifulco CB, Masucci G, Rau T (2016). Immunoscore and Immunoprofiling in cancer: an update from the melanoma and immunotherapy bridge 2015. J Transl Med.

[35] Topalian SL, Hodi FS, Brahmer JR, Gettinger SN, Smith DC (2012). Safety, activity, and immune correlates of anti-PD-1 antibody in cancer. N Engl J Med.

[36] Sunshine J, Taube JM (2015). PD-1/PD-L1 inhibitors. Curr Opin Pharmacol.

[37] Weber JS, Gibney G, Sullivan RJ, Sosman JA, Slingluff CL (2016). Sequential administration of nivolumab and ipilimumab with a planned switch in patients with advanced melanoma (CheckMate 064): an open-label, randomised, phase 2 trial. Lancet Oncol.

[38] Le DT, Uram JN, Wang H, Bartlett BR, Kemberling H (2015). PD-1 blockade in tumors with mismatch-repair deficiency. N Engl J Med.

[39] Powles T, Eder JP, Fine GD, Braiteh FS, Loriot Y (2014). MPDL3280A (anti-PD-L1) treatment leads to clinical activity in metastatic bladder cancer. Nature.

[40] Rizvi NA, Hellmann MD, Snyder A, Kvistborg P, Makarov V (2015). Cancer immunology. Mutational landscape determines sensitivity to PD-1 blockade in non-small cell lung cancer. Science.

[41] Rosenberg JE, Hoffman-Censits J, Powles T, van der Heijden MS, Balar AV (2016). Atezolizumab in patients with locally advanced and metastatic urothelial carcinoma who have progressed following treatment with platinum-based chemotherapy: a single-arm, multicentre, phase 2 trial. Lancet.

[42] Perou CM, Sorlie T, Eisen MB, van de Rijn M, Jeffrey SS (2000). Molecular portraits of human breast tumours. Nature.

[43] Sorlie T, Perou CM, Tibshirani R, Aas T, Geisler S (2001). Gene expression patterns of breast carcinomas distinguish tumor subclasses with clinical implications. Proc Natl Acad Sci USA.

[44] Kristensen VN, Lingjaerde OC, Russnes HG, Vollan HK, Frigessi A (2014). Principles and methods of integrative genomic analyses in cancer. Nat Rev Cancer.

[45] Fleischer T, Frigessi A, Johnson KC, Edvardsen H, Touleimat N (2014). Genome-wide DNA methylation profiles in progression to in situ and invasive carcinoma of the breast with impact on gene transcription and prognosis. Genome Biol.

[46] Aure MR, Jernstrom S, Krohn M, Vollan HK, Due EU (2015). Integrated analysis reveals microRNA networks coordinately expressed with key proteins in breast cancer. Genome Med.

[47] Wang S, Wu X, Chen Y, Zhang J, Ding J (2012). Prognostic and predictive role of JWA and XRCC1 expressions in gastric cancer. Clin Cancer Res.

[48] Xu W, Chen Q, Wang Q, Sun Y, Wang S (2014). JWA reverses cisplatin resistance via the CK2-XRCC1 pathway in human gastric cancer cells. Cell Death Dis.

[49] Christopher AF, Kaur RP, Kaur G, Kaur A, Gupta V (2016). MicroRNA therapeutics: discovering novel targets and developing specific therapy. Perspect Clin Res.

[50] Meister G (2013). Argonaute proteins: functional insights and emerging roles. Nat Rev Genet.

[51] Sempere LF (2014). Tissue slide-based microRNA characterization of tumors: how detailed could diagnosis become for cancer medicine?. Expert Rev Mol Diagn.

[52] Jorgensen S, Baker A, Moller S, Nielsen BS (2010). Robust one-day in situ hybridization protocol for detection of microRNAs in paraffin samples using LNA probes. Methods.

[53] Hansen TF, Kjaer-Frifeldt S, Christensen RD, Morgenthaler S, Blondal T (2014). Redefining high-risk patients with stage II colon cancer by risk index and microRNA-21: results from a population-based cohort. Br J Cancer.

[54] Nielsen BS, Jorgensen S, Fog JU, Sokilde R, Christensen IJ (2011). High levels of microRNA-21 in the stroma of colorectal cancers predict short disease-free survival in stage II colon cancer patients. Clin Exp Metastasis.

[55] Eriksen AH, Andersen RF, Nielsen BS, Sorensen FB, Appelt AL (2016). Intratumoral heterogeneity of microRNA expression in rectal cancer. PLoS ONE.

[56] Kirschner MB, Cheng YY, Armstrong NJ, Lin RC, Kao SC (2015). MiR-score: a novel 6-microRNA signature that predicts survival outcomes in patients with malignant pleural mesothelioma. Mol Oncol.

[57] Cleaver JE (1968). Defective repair replication of DNA in xeroderma pigmentosum. Nature.

[58] O’Connor MJ (2015). Targeting the DNA damage response in cancer. Mol Cell.

[59] Pettersen HS, Galashevskaya A, Doseth B, Sousa MM, Sarno A (2015). AID expression in B-cell lymphomas causes accumulation of genomic uracil and a distinct AID mutational signature. DNA Repair (Amst).

[60] Pao W, Miller V, Zakowski M, Doherty J, Politi K (2004). EGF receptor gene mutations are common in lung cancers from “never smokers” and are associated with sensitivity of tumors to gefitinib and erlotinib. Proc Natl Acad Sci USA.

[61] Lynch TJ, Bell DW, Sordella R, Gurubhagavatula S, Okimoto RA (2004). Activating mutations in the epidermal growth factor receptor underlying responsiveness of non-small-cell lung cancer to gefitinib. N Engl J Med.

[62] Cancer Genome Atlas Research N (2012). Comprehensive genomic characterization of squamous cell lung cancers. Nature.

